# Eosinophilic Colitis Associated with Emtricitabine/Tenofovir

**DOI:** 10.7759/cureus.3498

**Published:** 2018-10-26

**Authors:** Matthew R Lozier, Alexandra M Sanchez, Ricardo Reyes

**Affiliations:** 1 Internal Medicine, University of Miami, Holy Cross Hospital, Fort Lauderdale, USA

**Keywords:** eosinophilic gastroenteritis, hiv, emtricitabine/tenofovir, atopic disorders, eosinophils, crp, temporal association

## Abstract

Eosinophilic gastroenteritis (EGE) is a rare disease occurring commonly in patients with a history of allergies, asthma, food sensitivities, or parasite exposure. The condition has been postulated to occur when eosinophils accumulate at the intestinal epithelium resulting in inflammation, recruitment and degranulation of mast cells, and further propagation of eosinophil activation. While laboratory and microbiological data may point to the diagnosis, the condition is only confirmed via biopsy demonstrating the eosinophilic infiltration of the intestinal epithelium. Multiple medications have previously been implicated in causing EGE; however, this is the first documented case associated with emtricitabine/tenofovir.

## Introduction

Eosinophilic gastroenteritis (EGE) is a rare disease that is challenging to diagnose based on clinical findings, especially in patients without a history of allergies, asthma, food sensitivities, or parasite exposure. We report a biopsy-confirmed case of EGE likely related to treatment with the combination of emtricitabine/tenofovir. Repeat administration of the drug resulted in abdominal symptoms and imaging findings consistent with colitis, which resolved following the cessation of therapy.

## Case presentation

**Figure 1:**
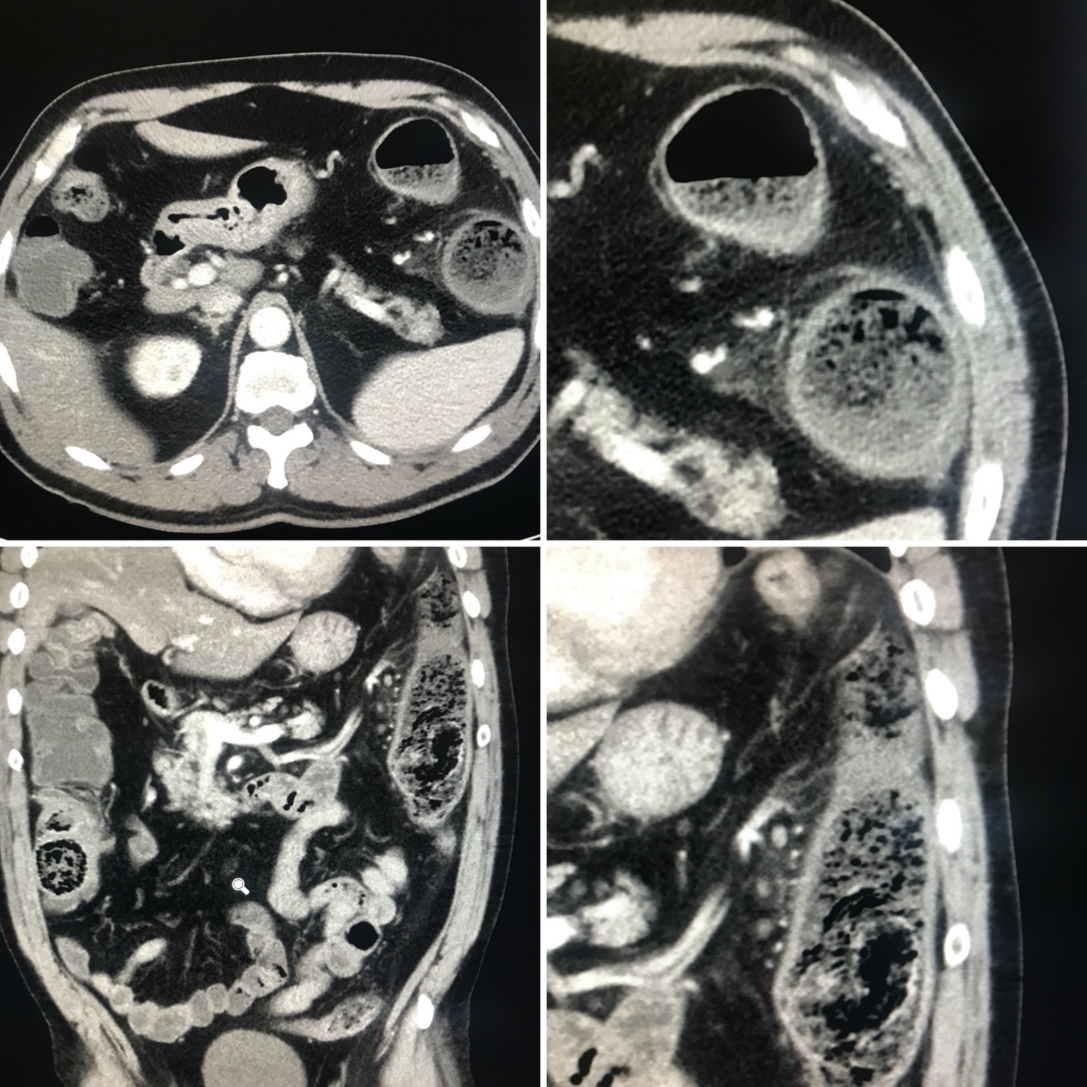
Focal colitis Axial (top) and coronal (bottom) views of the computed tomography scan of the abdomen with evidence of fecalith in the proximal and mid-descending colon associated with focal colonic wall thickening, pericolonic inflammatory stranding, and pericolonic fluid consistent with focal colitis

**Figure 2:**
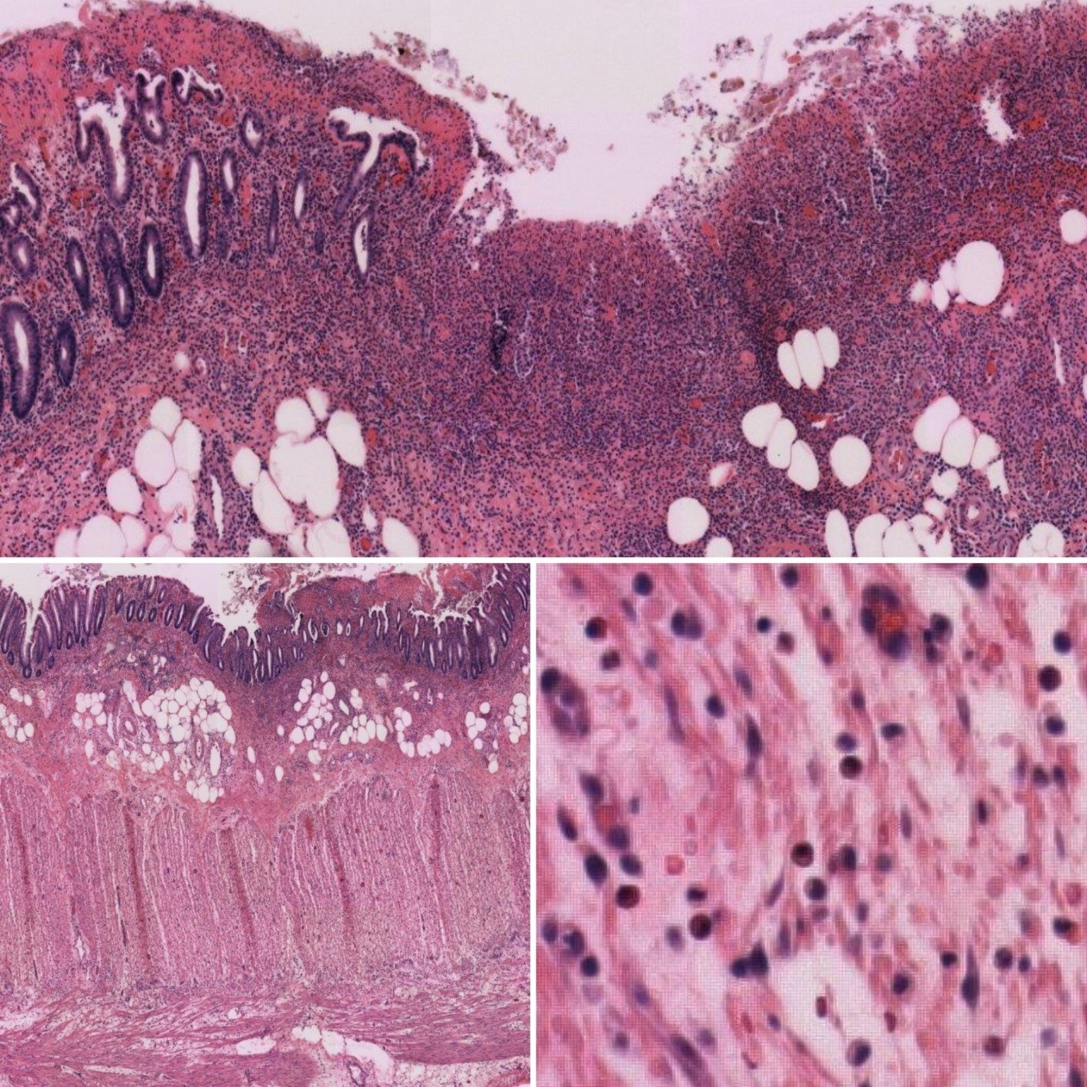
Pathologic findings Low-power microscopic views (top and lower left) of a pathologic specimen of the colon demonstrating numerous eosinophils infiltrating throughout. High-power microscopic view (lower right) demonstrating similar findings

A 54-year-old male with previously diagnosed human immunodeficiency virus (HIV) had recurrent presentations for colitis since initiating emtricitabine/tenofovir. In the past, he had self-discontinued this medication, resulting in the resolution of gastrointestinal (GI) complaints, but subsequent re-initiation of the medication led to a recurrence of symptoms and hospitalization. The abdominal computed tomography (CT) scan reported focal colitis in the descending colon, as seen in Figure 1. The patient was started on empiric antibiotics with a progressive worsening of symptoms. He was taken to the operating room for exploratory laparoscopy, resulting in colonic resection and diverting colostomy. The pathological specimen, as seen in Figure 2, demonstrated eruptive pseudomembranes, edema, and mixed inflammation, including numerous eosinophils within the colon wall consistent with eosinophilic colitis. Upon reviewing his laboratory and microbiological data, it was noted that the patient did not have peripheral eosinophilia and that stool cultures, fecal leukocytes, and stool ova and parasites were all negative. However, an elevated C-reactive protein (CRP) of 37.6 mg/L was noted on admission that trended up to 61.0 mg/L. Outpatient screening for HLA-B*57:01 was negative and his previous HIV medication regimen was switched to the combination of abacavir, dolutegravir, and lamivudine. No symptom recurrence has been noted since the treatment regimen was adjusted.

## Discussion

The diagnosis of EGE is based on the presence of eosinophilic infiltration of the stomach, duodenum, esophagus, and colon without any known cause of eosinophilia [1]. While peripheral eosinophilia is a common finding in EGE, it is not necessary for the diagnosis [2]. The pathogenesis of EGE is not well understood, but epidemiologic and clinical features suggest an allergic component. It has been postulated that eosinophils accumulate at the intestinal surface and release multiple toxic cationic proteins. These proteins cause the destruction of the intestinal epithelium, resulting in recruitment and degranulation of mast cells, further propagating eosinophil activation [3]. This case of EGE is unique, as the patient had no history of allergies or atopic disorders, no peripheral eosinophilia, and negative microbiological tests (stool cultures, fecal leukocytes, and stool ova and parasites). Even with all these unremarkable findings, the biopsy specimen demonstrated marked eosinophilic infiltration of the mucosal layers. While other possible causes (allergic, inflammatory, parasitic infection, and more) of EGE were considered, the temporal association of GI symptoms matching the use of emtricitabine/tenofovir and resolution following the adjustment of the HIV regimen implicate this medication as the cause in this clinical scenario. Although multiple medications have previously been implicated in causing EGE [3-9], this is the first documented case associated with emtricitabine/tenofovir.

## Conclusions

This case should raise awareness that there are more undocumented medications that can cause EGE. We propose that EGE be considered in the differential diagnosis of patients taking emtricitabine/tenofovir who develop recurring gastrointestinal symptoms and have imaging findings consistent with colitis even if peripheral eosinophilia is not noted. Colonoscopy and biopsy may be warranted during the evaluation in order to definitively diagnose or rule out the disease.

## References

[1] Uppal V, Kreiger P, Kutsch E (2016). Eosinophilic gastroenteritis and colitis: a comprehensive review. Clin Rev Allergy Immunol.

[2] Talley NJ, Shorter RG, Phillips SF, Zinsmeister AR (1990). Eosinophilic gastroenteritis: a clinicopathological study of patients with disease of the mucosa, muscle layer, and subserosal tissues. Gut.

[3] Lee JY, Medellin MV, Tumpkin C (2000). Allergic reaction to gemfibrozil manifesting as eosinophilic gastroenteritis. South Med J.

[4] Shouval R, Duffield A, Gocke C, Lee L, Brodsky RA (2019). Pentostatin-induced hypereosinophilia with eosinophilic gastroenteritis. Leuk Lymphoma.

[5] Barak N, Hart J, Sitrin MD (2001). Enalapril-induced eosinophilic gastroenteritis. J Clin Gastroenterol.

[6] Wienand B, Sanner B, Liersch M (1991). Eosinophilic gastroenteritis as an allergic reaction to a trimethoprim-sulfonamide preparation [Article in German, English]. Dtsch Med Wochenschr.

[7] Anttila VJ, Valtonen M (1992). Carbamazepine-induced eosinophilic colitis. Epilepsia.

[8] Lange P, Oun H, Fuller S, Turney JH (1994). Eosinophilic colitis due to rifampicin. Lancet.

[9] Karmacharya R, Mino M, Pirl WF (2005). Clozapine-induced eosinophilic colitis. Am J Psychiatry.

